# Type 2 Diabetes, Atrial Fibrillation, and Direct Oral Anticoagulation

**DOI:** 10.1155/2019/5158308

**Published:** 2019-12-06

**Authors:** Dana Prídavková, Matej Samoš, Tomáš Bolek, Ingrid Škorňová, Jana Žolková, Peter Kubisz, Ján Staško, Marián Mokáň

**Affiliations:** ^1^Department of Internal Medicine I, Jessenius Faculty of Medicine in Martin, Comenius University in Bratislava, Martin, Slovakia; ^2^National Center of Hemostasis and Thrombosis, Department of Hematology and Blood Transfusion, Jessenius Faculty of Medicine in Martin, Comenius University in Bratislava, Martin, Slovakia

## Abstract

Type 2 diabetes (T2D) is an independent risk factor of stroke and systemic embolism in patients with atrial fibrillation (AF), and T2D patients with AF-associated stroke seem to have worse clinical outcome and higher risk of unfavorable clinical course compared to individuals without this metabolic disorder. Long-term anticoagulation is indicated in majority of T2D patients with AF to prevent adverse AF-associated embolic events. Direct oral anticoagulants (DOACs), direct oral thrombin inhibitor dabigatran, and direct oral factor Xa inhibitors, rivaroxaban, apixaban, and edoxaban, have emerged as a preferred choice for long-term prevention of stroke in AF patients offering potent and predictable anticoagulation and a favorable pharmacology with low risk of interactions. This article reviews the current data regarding the use of DOACs in individuals with T2D and AF.

## 1. Introduction

Type 2 diabetes (T2D) is an independent risk factor of stroke and systemic embolism in patients with atrial fibrillation (AF). Moreover, T2D patients with AF-associated stroke seem to have worse clinical outcome and higher risk of unfavorable clinical course compared to individuals without this metabolic disorder [1–4]. Long-term anticoagulation to prevent stroke or systemic embolism is indicated in the majority of AF patients depending on individual estimation of risk for these adverse events [5]. Considering the fact that the prevalence of hypertension and vascular diseases is high among T2D patients, there is a high probability that T2D patients with newly onset AF would require long-term anticoagulation for prevention of future embolic events. Direct oral anticoagulants (DOACs), direct oral thrombin inhibitor dabigatran, and direct oral factor Xa inhibitors, rivaroxaban, apixaban, and edoxaban, have emerged as a preferred choice for long-term prevention of stroke in AF patients [5] offering potent and predictable anticoagulation and a favorable pharmacology with low risk of interactions. Now, the question is of whether T2D affects the efficacy and/or safety of long-term anticoagulation with DOACs. This article reviews the current data regarding the use of DOACs in individuals with T2D and AF.

## 2. T2D, Prevalence of AF, and the Risk of Stroke and Systemic Embolism in the Settings of AF

AF is quite frequent as heart arrhythmia in individuals with T2D, and several studies suggested that AF is more frequent in T2D patients compared to those without T2D. Ostgren et al. [6] showed in their community-based, cross-sectional observation that the age-adjusted prevalence of AF was the highest among patients with T2D and hypertension (6%), compared to 4% in patients with T2D only and to 2% in controls, respectively. Furthermore, T2D is an independent contributor to increased prevalence and incidence of AF. This fact was demonstrated in an observational cohort study enrolling totally 17,372 patients from HMO Diabetes registry and matching them to patients without diabetes [7]. In the studied population, diabetes was an independent determinant of AF prevalence (3.6% in diabetic patients versus 2.5% in individuals without diabetes, *p* < 0.0001), and predicted the incidence of AF among women. Over a mean follow-up of 7.2 ± 2.8 years, diabetic patients without AF at baseline developed AF at an age- and sex-adjusted rate of 9.1 per 1,000 person-years compared to a rate of 6.6 among nondiabetic individuals. Similar observations were published from the Atherosclerosis Risk in Communities study [8]. In this study, enrolling 13,025 individuals, T2D, HbA1c levels, and poor glycemic control were independently associated with an increased risk of AF. Moreover, a meta-analysis of 11 studies with data from 108,703 cases of AF in 1,686,097 subjects confirmed that diabetes is associated with an increased risk of subsequent AF [9]. Now, it is not entirely clear what is/are the underlying mechanism/mechanisms responsible for such a high prevalence of AF in T2D patients or whether there is a direct association between these disorders. Although a direct association between T2D and AF remains so far speculative, several mechanisms, such as T2D-associated atrial structural, mechanical and electrical remodeling, diabetic autonomic neuropathy, endothelial dysfunction, proinflammation, activation of renin-angiotensin system, and T2D-associated pathologic angiogenesis, directly linking these entities have been previously reported [10]. Another question is whether the risk of AF can be affected with a therapeutic approach, such as intensive/tight/glycemic control. This question was examined in a post hoc analysis of a randomized, double-blind fashion trial Action to Control Cardiovascular Risk in Diabetes (ACCORD), which aimed to prospectively evaluate if intensive glycemic control in patients with diabetes affects the incidence of AF and to evaluate morbidity and mortality in patients with DM and incident AF [11]. This trial randomized 10,082 T2D patients to intensive therapeutic strategy (with a target HbA1c of <6%) or a standard strategy (HbA1c of 7.0 to 7.9%), and followed the patients for a median of 3.4 years. In this study, intensive glycemic control did not affect the rate of new-onset AF. However, patients with T2D and incident AF had an increased risk for morbidity and mortality compared with those without AF. On the other hand, several other studies suggested that the risk of incident AF in T2D patients could be reduced with intensive blood pressure lowering therapy [12], biguanides (metformin) and thiazolidinediones [13], or dipeptidyl peptidase-4 (DPP-4) inhibitor therapy [14]. Summarizing, although there is strong evidence that T2D increases the risk of new-onset AF, and that this risk seems to be even higher in women with T2D [7, 15], the mechanisms responsible for this higher risk would have to be explored in future studies, and there is still a gap in evidence of whether this risk could be modified with a pharmacologic/nonpharmacologic approach.

Additionally, T2D seems to be connected with an increased risk of AF-induced stroke/systemic embolism, and AF-induced stroke in T2D patients is associated with worse clinical outcome and higher risk of stroke-related complications compared to nondiabetic AF patients. Klem et al. [16] showed in their prospective study with ten years of follow-up in patients with AF that T2D patients with AF had larger left atria and left atrial appendages, had more frequent left atrial or appendage thrombi, and had higher mortality (7%/year versus 4%/year, *p* < 0.0001) than nondiabetic patients. Although the rate of stroke or embolism in this particular study did not differ significantly between T2D (3%/year) and nondiabetic patients, the rate of oral anticoagulation was higher in T2D patients than in nondiabetic ones. In addition, another previously published study with 464 T2D patients and matched healthy subjects [17] demonstrated that subclinical episodes of AF were significantly more frequent among patients with T2D compared with matched healthy individuals (11% versus 1.6%, *p* < 0.0001). Moreover, in this study, these subclinical episodes of AF were associated with a significantly increased risk of silent cerebral infarction and symptomatic stroke. Therefore, there is a strong probability that the presence of AF itself predicts future vascular brain disease and stroke in T2D patients. Furthermore, the incidence of hospitalization due to stroke is higher in those without T2D; T2D seems to be positively associated with ischemic stroke in men and women and seems to be related to higher in-hospital mortality and readmission rates especially in women with T2D [18]. In a retrospective analysis of 286 consecutive cases of ischemic stroke [19], T2D (specially unknown T2D) was identified as a strong risk factor for in-hospital mortality. Patients with unknown T2D showed more severe neurological damage (measured using Canadian Neurological Score) than subjects with known T2D and nondiabetic subjects. Now, the question might be of whether the risk of ischemic stroke correlates with the metabolic control of T2D. The analysis of Danish registries [20] suggested that the risk of AF-associated thromboembolism seems to be higher in T2D patients with poorer metabolic control of the disease (with higher levels of HbA1c). Compared with patients with HbA1c ≤ 48 mmol/mol, there was a higher risk of embolism among patients with HbA1c = 49‐58 mmol/mol (hazard ratio: 1.49; 95% CI: 1.09-2.05) and HbA1c > 58 mmol/mol (hazard ratio: 1.59; 95% CI: 1.13-2.22). However, in this analysis, no such association was found in patients with T2D duration of ≥10 years. This observation was not fully explained. Finally, T2D and previous AF were identified in a fully adjusted multivariable logistic regression analysis as significant risk factors for ischemic stroke also in individuals who developed their first event of stroke at the age of 25 to 49 years [21]. This observation suggests that T2D patients with AF are at a higher risk of early-onset stroke compared with nondiabetic individuals.

Embolic stroke is a devastating complication of AF, possibly leading to long-term disability, with life-long dependency or a need for institutionalization in a nursing home, or even to death [22]. Long-term oral anticoagulation is indicated in majority of AF patients to reduce the risk of future ischemic adverse events [5], i.e., to reduce the risk of embolic stroke or systemic embolism. As already mentioned, the prevalence of high blood pressure, vascular diseases, left atrial enlargement, and left ventricular dysfunction is higher in T2D AF patients compared to nondiabetic ones [16]. Thus, the majority of T2D patients with AF would require long-term oral anticoagulation. Vitamin K antagonist (VKA) therapy has been used for oral anticoagulation in AF patients for decades. Although no reduced efficacy or no safety concerns regarding the use of VKA anticoagulation in T2D patients have been so far reported, T2D was previously associated with increased therapeutic (international normalized ratio (INR)) variability in Chinese patients with AF [23]. Furthermore, VKA therapy is connected with other disadvantages, mostly the need for frequent laboratory monitoring and dose adjustment, long-lasting anticoagulant activity, and the risk of unexpected drug and food interactions; therefore, achieving and maintaining the optimal therapeutic activity of VKA might be extremely difficult [24]. Considering the fact that T2D patients usually require poly-pharmacotherapy not only to treat T2D but also to treat T2D-related diseases and complications [25], there is a particular risk of VKA therapy affecting drug interactions in individuals with T2D. On the other side, direct (novel, non-vitamin K-dependent) oral anticoagulants (DOACs) offer several advantages, such as more predictable inhibition of coagulation compared to VKA, more rapid on- and offset of action, shorter plasma half-life, lower risk of food and drug interactions, and no need for a routine laboratory monitoring of achieved anticoagulant activity. Due to these advantages, DOACs should be recently preferred for long-term oral anticoagulation in AF patients [5]. Now, the question is: do we have sufficient data on the efficacy and safety of DOACs in T2D patients with AF?

## 3. T2D and Long-Term Dabigatran Therapy for AF

**Figure 1:**
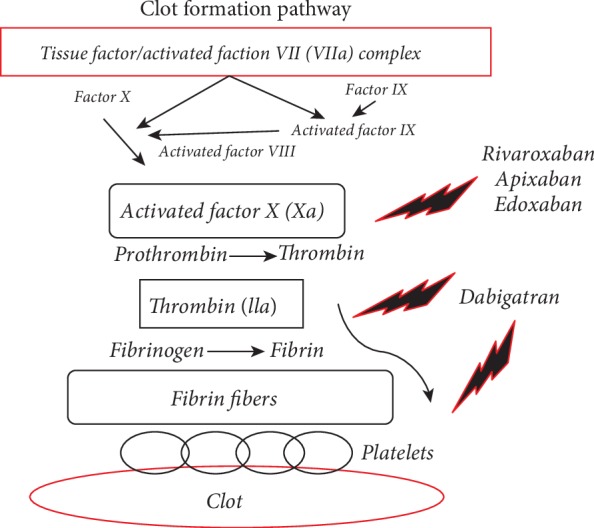
Clot formation pathway and the mechanism of action of direct oral anticoagulants.

Dabigatran etexilate [26] is the first DOAC introduced for the prevention of stroke and systemic embolism in AF patients by the RE-LY (Randomized Evaluation of Long-Term Anticoagulation Therapy) study [27]. Acting as direct thrombin (factor IIa) inhibitor (inhibiting both free and platelet bond thrombin), dabigatran rapidly inhibits coagulation (Figure 1) and might also affect (reduce) thrombin-induced platelet aggregation [28, 29]. Dabigatran etexilate (Table 1) is after its absorption (which is probably pH dependent) rapidly converted to an active form—dabigatran by esterases. Dabigatran is a substrate for P-glycoprotein (P-gp); but its metabolism is cytochrome P (CYP) 450 independent. It has a plasma half-life of 14–17 hours (in individuals with normal kidney functions) and is eliminated mostly by the kidneys, mostly through glomerular filtration. Considering these data, the change of P-gp transport capacity, reduced glomerular filtration, and change in gastric pH (for example, during concomitant coadministration of proton pump inhibitors) might change dabigatran half-life, its plasma levels, and its activity [26, 30]. In addition, previously published studies showed that diabetes itself could affect the P-gp transport capacity [31]; therefore, there is a theoretic possibility that the efficacy of dabigatran therapy could be changed in T2D patients.

Truth of the matter, looking more closely on the data about the efficacy and safety of dabigatran in T2D patients, the information is still very limited, in fact, according to a subanalysis of the RE-LY study [32] and several postmarketing data [33, 34]. A clinical efficacy and safety data on dabigatran therapy in T2D patients from the RE-LY trial were reported in a subanalysis of this trial [32]. Of 18,113 patients totally enrolled in the trial, 4,221 patients had T2D (23.3%). The results of this subanalysis (Table 2) showed that the absolute reduction in stroke or systemic embolism with dabigatran compared to warfarin was greater among patients with T2D than that in patients without diabetes (0.59% per year versus 0.05% per year for dabigatran 110 mg twice daily; 0.89% per year versus 0.51% per year for dabigatran 150 mg twice daily, respectively). However, it is necessary to add that regardless of treatment strategy (VKA or dabigatran), adverse embolism was more common among patients with T2D (1.9% per year versus 1.3% per year, *p* < 0.001). T2D was also associated with an increased risk of death (5.1% per year versus 3.5% per year, *p* < 0.001) and major bleeding (4.2% per year versus 3.0% per year, *p* < 0.001). The risk of major bleeding in T2D patients was similar in both dabigatran groups and in the warfarin group (4.66% for dabigatran 150 mg twice daily versus 3.81% for dabigatran 110 mg twice daily versus 4.19% for warfarin). However, the number of T2D patients who experienced intracranial bleeding was significantly lower in patients treated with 110 mg of dabigatran twice daily compared to those receiving warfarin. The authors of this subanalysis concluded that in the RE-LY study, compared to nondiabetic patients, T2D patients with AF derived a greater absolute risk reduction in embolic events when treated with dabigatran.

Similar results regarding the efficacy and safety of dabigatran in diabetic patients were subsequently published from the analysis of the nationwide diabetes program in Taiwan [33]. The authors of this analysis identified 322 diabetic AF patients on dabigatran and compared them with 1,899 diabetic AF patients on warfarin. In this analysis, compared with warfarin, dabigatran significantly decreased the risk of all-cause mortality (hazard ratio = 0.348, 95% confidence interval = 0.157‐0.771) and gastrointestinal bleeding (hazard ratio = 0.558, 95% confidence interval = 0.327‐0.955). Furthermore, the effect of T2D on DOACs' plasma levels/activity (including dabigatran) in patients with AF was specifically examined in our previous pilot prospective study. In this study, we measured trough and peak plasma levels with Hemoclot® Thrombin Inhibitor Assay and found no significant differences in these levels comparing T2D patients with nondiabetic individuals [34]. This observation suggests that T2D is probably not associated with the risk of change in dabigatran plasma levels in AF patients; however, up-to-date, there is no study for the confirmation of these results, and due to possible limitations of our analysis, these results should be interpreted still with caution. Summarizing the data, recently, the data suggest that, in diabetic AF patients, dabigatran showed a superior efficacy and a comparable (or even superior) safety profile compared with warfarin and T2D probably does not change the on-treatment levels in dabigatran-treated patients. Nevertheless, dabigatran cannot be used in patients with moderate and severe reduction of kidney functions, as several cases of serious overdose/life-threatening bleeding have been reported in individuals with reduce kidney function or acute kidney injury [35–38]. This would probably limit the use of dabigatran in T2D patients (as there is a high prevalence of diabetic kidney disease in these patients).

## 4. T2D and Long-Term Rivaroxaban Therapy for AF

Rivaroxaban [39], the second DOAC approved for AF patients on the basis of the results of the ROCKET AF (The Rivaroxaban One Daily Oral Direct Factor Xa Inhibition Compared with Vitamin K Antagonism for Prevention of Stroke and Embolism Trial in Atrial Fibrillation) trial [40], acts as a direct oral factor Xa inhibitor (FXaI) (Figure 1). Rivaroxaban (Table 1) exposes rapid absorption, has high bioavailability, binds to plasma protein, and is eliminated unchanged by the kidneys or after metabolic transformation by CYP and degradation by the kidneys and by the hepatobiliary system. Rivaroxaban has a plasma half-life of 5–13 hours and has a low number of food and drug interactions. However, its activity might be affected with strong induction/inhibition of CYP enzymes or due to severe reduction of the kidney functions [39]. Rivaroxaban probably does not affect platelet aggregation [41, 42]. Several previously published studies [43–45] pointed on the fact that diabetes modulates the activity of CYP enzymes; hence, this modulation can, in theory, lead to changed rivaroxaban activity in T2D subjects.

The efficacy and safety of rivaroxaban in T2D patients was evaluated in the subanalysis of ROCKET AF trial [46]. This trial included 5,695 diabetic patients (40%). The subanalysis of this trial (Table 2) demonstrated that the relative efficacy of rivaroxaban and warfarin for the prevention of stroke and systemic embolism was similar in patients with (1.74 versus 2.14/100 patient-years, hazard ratio: 0.82) and without (2.12 versus 2.32/100 patient-years, hazard ratio: 0.92) T2D (*p* = 0.53). Looking on the safety profile, the safety of rivaroxaban versus warfarin regarding major bleeding (hazard ratios: 1.00 and 1.12 for patients with and without T2D, respectively, *p* = 0.43), major or nonmajor clinically relevant bleeding (hazard ratios: 0.98 and 1.09, *p* = 0.17), and cerebral hemorrhage (hazard ratios: 0.62 and 0.72, *p* = 0.67) was independent of T2D. However, in an adjusted exploratory analysis, T2D patients had 1.3-, 1.5-, and 1.9-fold higher 2-year rates of stroke, vascular mortality, and myocardial infarction than nondiabetic ones. Summarizing, in the ROCKET AF trial, the relative efficacy and safety of rivaroxaban versus VKA warfarin was similar in patients with and without T2D.

Peacock et al. [47] studied the impact of T2D on the incidence of major bleeding in rivaroxaban-treated patients with AF. Among the 44,793 rivaroxaban users enrolled in this analysis, 26.9% had T2D (12,039 patients). In this analysis, major bleeding incidence was higher among those with T2D compared with those without (3.68 versus 2.51 per 100 person-years), and intracranial bleeding incidence was 0.19 versus 0.25 per 100 person-years. Fatal outcomes were rare for both cohorts. Therefore, in this particular analysis, T2D AF patients treated with rivaroxaban had higher incidence of major bleeding compared to nondiabetic ones. On the other side, previously mentioned analysis of the diabetes program in Taiwan [33] reported comparable efficacy and safety outcomes in rivaroxaban-treated and warfarin-treated T2D patients. In addition, in our pilot prospective study [34] examining the impact of T2D on therapeutic activity of rivaroxaban using rivaroxaban-calibrated anti-Xa chromogenic analysis, there were no significant differences in rivaroxaban trough and peak anti-Xa activity comparing T2D patients and patients without T2D. Although the limitations of the analysis should be considered, this observation suggests that T2D probably does not affect the therapeutic activity in rivaroxaban-treated patients with AF. Additionally, another postmarketing analysis of 5,517 rivaroxaban users and 5,517 warfarin users with AF and T2D [48] showed that rivaroxaban was associated with nonsignificant reductions in stroke or systemic embolism (0.87 versus 1.35/100 person-years; hazard ratio: 0.68, 95% confidence interval: 0.44-1.05) compared with warfarin. Furthermore, no differences in major bleeding (2.7 versus 3.0/100 person-years; hazard ratio: 0.96, 95% confidence interval: 0.74-1.25) were observed. In another prospective study enrolling patients with T2D and AF treated with rivaroxaban (10,700 patients) and warfarin (13,946), Baker et al. [49] reported that rivaroxaban therapy was associated with a 25% reduced risk of major adverse cardiac events and a 63% reduced risk of major adverse limb events compared to warfarin; major bleeding risk did not significantly differ between rivaroxaban- and warfarin-treated patients with T2D. Finally, a recent analysis [50] of 10,017 rivaroxaban and 11,665 warfarin users with T2D and AF suggested that rivaroxaban appears to be associated with lower risks of undesirable renal outcomes versus warfarin in diabetic AF patients. In this analysis, rivaroxaban was associated with lower risks of acute kidney injury (hazard ratio = 0.83, 95% confidence interval = 0.74–0.92) and development of stage 5 chronic kidney disease or need for hemodialysis (hazard ratio = 0.82, 95% confidence interval = 0.70–0.96). Summarizing the data regarding the use of rivaroxaban in T2D patients, current evidence is in favor of comparable (or even superior) efficacy of rivaroxaban in T2D patients with AF and comparable safety profile of rivaroxaban and VKA therapy in these patients.

## 5. T2D and Long-Term Apixaban Therapy for AF

Apixaban [51] is the next DOAC approved for the prevention of embolism in AF patients on the basis of the results reported from the ARISTOTLE (Apixaban for Reduction in Stroke and Other Thromboembolic Events in Atrial Fibrillation) trial [52, 53]. Apixaban acts as a direct oral FXaI (Figure 1). Apixaban (Table 1) has a favorable pharmacologic profile, with linear pharmacokinetics, food-independent absorption, and small distribution volume (0.3 l/kg). Apixaban achieves its maximal plasma levels in 3–4 hours after ingestion, with a mean elimination half-life of 8–15 hours. There are no relevant age- or sex-dependent differences in apixaban pharmacologic profile. Apixaban is metabolized by CYP (especially by CYP 3A4) and is also a substrate for the P-gp, and its elimination is mediated by the metabolism and kidneys and its excretion in the gastrointestinal tract [51]. Considering these data, strong inducers or strong inhibitors of CYP and P-gp might affect the plasma levels and activity of apixaban. In addition, apixaban might be safely used in adults with end-stage kidney disease [52, 53], probably does not affect platelet aggregation [41, 42], and there is no relevant interaction between proton pump inhibition and apixaban [54]. As already mentioned, T2D might affect both P-gp and CYP activity [31, 43–45]; thus, there is a theoretic possibility that apixaban levels and/or activity might be changed in T2D patients.

In fact, currently available data regarding the use of apixaban in T2D patients are limited to a subanalysis of the ARISTOTLE trial [55] and our previous analysis of the impact of T2D on apixaban plasma activity in AF patients [34]. A subanalysis of the efficacy and safety of apixaban versus warfarin in T2D patients with AF (4,547 patients = 24.9% of patients) from the ARISTOTLE trial [55] showed that apixaban-treated patients with T2D had lower rates of stroke and systemic embolism (hazard ratio: 0.75, 95% confidence interval: 0.53-1.05), all-cause mortality (hazard ratio: 0.83, 95% confidence interval: 0.67-1.02), cardiovascular mortality (hazard ratio: 0.89, 95% confidence interval: 0.66-1.20), intracranial hemorrhage (hazard ratio: 0.49, 95% confidence interval: 0.25-0.95), and a similar rate of myocardial infarction (hazard ratio: 1.02, 95% confidence interval: 0.62-1.67) compared with warfarin. For major bleeding, a quantitative interaction was seen (*p* = 0.003) with a greater reduction in major bleeds in patients without T2D even after multivariable adjustment (Table 2). Therefore, in this subanalysis, apixaban had similar benefits on reducing stroke, decreasing mortality, and causing less cranial bleeds than warfarin in patients with and without T2D. Additionally, in our previous study using apixaban-calibrated anti-Xa chromogenic analysis [34], there were no significant differences in apixaban trough 96.0 ± 54.5 versus 63.9 ± 36.8 ng/ml, *p* = 0.24) and peak (151.0 ± 78.3 versus 151.7 ± 59.1 ng/ml, *p* = 0.98) anti-Xa activity between T2D and nondiabetic AF patients, implicating no relevant impact of T2D on therapeutic activity of apixaban. Thus, although currently available data are very limited, it seems that apixaban achieves in T2D patients with AF similar efficacy, safety, and therapeutic activity than in nondiabetic ones. Nevertheless, further studies will probably be needed for the confirmation of this statement.

## 6. T2D and Long-Term Edoxaban Therapy for AF

Edoxaban [56] is the latest approved oral FXaI (Figure 1) for the prevention of embolic events in patients with AF. Prior its approval, edoxaban was tested in a randomized ENGAGE AF-TIMI 48 (Effective Anticoagulation with Factor Xa Next Generation in Atrial Fibrillation-Thrombolysis in Myocardial Infarction 48) trial [57]. Edoxaban (Table 1) has a rapid absorption, a linear pharmacokinetics, with maximal plasma levels achieved at 1.5 hours after oral administration, has a half-life of 10–14 hours, undergoes biotransformation mostly through hydrolysis, has a CYP-independent metabolism, but is a substrate for P-gp. Edoxaban is eliminated through the hepatobiliary system (60%) and the kidneys. An increased edoxaban exposure was reported when edoxaban was coadministrated with amiodarone, dronedarone, quinidine, and verapamil [56]. There is a possible T2D/edoxaban interaction which could be, in theory, mediated trough T2D/P-gp interaction.

Unfortunately, in contrast to dabigatran, rivaroxaban, and apixaban, there is no subanalysis of phase III clinical trial or no postmarketing study reporting the efficacy and safety data among AF patients with T2D. Although the ENGAGE AF-TIMI trial [57] enrolled patients with diabetes (in total 7,624 diabetic patients from 21,105 patients enrolled), the clinical outcomes of diabetic patients from this trial were not specifically reported. However, in the basic subgroup analysis [57], there were no significant differences in primary efficacy endpoints (hazard ratio: 1.42 versus 1.52, *p* = 0.54, for high-dose edoxaban versus warfarin and hazard ratio: 1.9 versus 1.52, *p* = 0.35, for low-dose edoxaban versus warfarin, respectively) or in primary safety endpoints (hazard ratio: 3.06 versus 3.94, *p* = 0.70, for high-dose edoxaban versus warfarin and hazard ratio: 1.74 versus 3.94, *p* = 0.52, for low-dose edoxaban versus warfarin, respectively) between T2D and nondiabetic patients (Table 2). Furthermore, no postmarketing study with edoxaban in T2D patients with AF is recently available, or undergoing. Thus, up-to-date, there are no exact data regarding the efficacy and safety of edoxaban in the subpopulation of T2D patients. Considering abovementioned data regarding the prevalence of AF in T2D patients, undoubtedly, there is a need for such subanalysis or prospective study in the near future.

## 7. Unanswered Issues and Practical Considerations regarding the Use of DOACs in T2D Patients with AF

First, there is an issue regarding unexplored drug interactions between DOACs and antidiabetic agents. As mentioned, T2D patients usually require multiple pills daily to treat T2D and/or T2D-related diseases and complications [25]. In fact, there are really insufficient data regarding the DOACs' pharmacology, efficacy, and safety when DOACs are coadministrated with antidiabetic agents. Although DOACs exhibit more favorable pharmacologic profile with lower risk of drug interactions [26, 39, 51, 56], and the metabolic profile of most frequently used antidiabetic agents does not assume such interactions [58], in fact, there is no pharmacokinetic/pharmacodynamic or clinical study examining the effect of metformin on dabigatran and oral FXaI activity. Thereinafter, no such study was performed for sulphonylureas, glucagon-like peptide-1 agonists, DPP-4 inhibitors, sodium glucose co-transporter-2 inhibitors, and insulin. Additionally, very limited data on these possible interactions exist for drugs so frequently used for the treatment of T2D-related diseases and complications, such as angiotensin-converting enzyme inhibitors [59] and statins [60, 61]. Therefore, this is the first issue which remains open for future research.

Second, another issue regarding the use of DOACs in T2D patients with diabetic kidney disease and reduced kidney function is not well explained. Looking more closely at this issue, the prevalence of diabetic kidney disease, defined as reduced glomerular filtration rate (GFR) or albuminuria or both, is generally high. A cross-sectional analysis [62] of adults with diabetes participating in National Health and Nutrition Examination Surveys in United States from 1988 to 2014 showed that the prevalence of diabetic kidney disease varied from 28.4% in 1988–1994 to 26.2% in 2009–2014. In this analysis, the prevalence of reduced GFR increased from 9.2% in 1988–1944 to 14.1% in 2009–2014. Another cross-sectional survey in Chinese rural residents (including 23,869 participants) demonstrated that the overall prevalence of chronic kidney disease in participants with diabetes was 35.5% [63]. Additionally, the high prevalence of abnormal kidney function among diabetic patients has been consistently reported in populations living in the United States [62], United Arab Emirates [64], Japan [65], Palestine [66], China [63, 67], Taiwan [68], and Europe [69–71]. Moreover, T2D probably predicts progression of chronic kidney disease, and the presence of diabetic kidney disease seems to be associated with poor cardiovascular risk profile, especially in elderly individuals [71, 72]. Furthermore, in accordance with DOACs' pharmacology, patients with severe renal impairment were excluded from phase III clinical trials with DOACs [27, 40, 52, 53, 57]. Considering these data, the fact that T2D patients might have reduced GFR, or might be in higher risk for acute worsening of kidney function, should be always considered carefully when a decision on long-term anticoagulation strategy is made. Now, it is necessary to say that there is no study evaluating the efficacy and safety of DOACs in a population of patients with known diabetic kidney disease. Thus, the efficacy/safety data for the use of DOACs in T2D patients with reduced GFR can be drawn only from studies on samples of unselected patients with kidney insufficiencies. In this population of patients with reduced GFR (of unselected etiology), in general, DOACs seem to be associated with lower risk of stroke in patients with mild and moderate renal impairment, as well as with fewer major bleeds among patients with mild and moderate kidney dysfunction [73]. Nevertheless, the safety concerns must be probably questioned especially in case of dabigatran long-term anticoagulation, as there are repeated reports of life-threatening dabigatran-induced bleeding in patients with acute kidney failure or acute kidney injury [36, 38, 74]. Generally, standard dabigatran (150 mg twice daily) can be safely administrated if GFR is above 50 ml/min/1.73 m^2^, and reduced dabigatran regimen (110 mg twice daily) is needed in patients with GFR 50–30 ml/min/1.73 m^2^. Nevertheless, dabigatran cannot be started and should be discontinued when GFR falls below 30 ml/min/1.73 m^2^. Although the U.S. Food and Drug Administration recently approved dabigatran 75 mg twice daily for patients with AF and severely impaired kidney function (GFR 15-30 ml/min/1.73 m^2^), and a pharmacokinetic/pharmacodynamic study (enrolling 60 treated subjects) showed that dabigatran 75 mg twice daily exposure levels in these patients largely confirmed earlier pharmacokinetic and pharmacodynamic predictions [75], supporting the use of dabigatran 75 mg twice daily in patients with AF and severe renal dysfunction, this approach can be considered just in the United States but cannot be used in other countries worldwide (in these countries, dabigatran dosed 75 mg twice daily can be used only to prevent venous thromboembolism in patients undergoing orthopedic surgery). Similarly, in a previous pharmacokinetic study [76], the increased area under the curve (AUC) value in patients with severe renal dysfunction was greater than expected also in the case of rivaroxaban, indicating that there might be a risk of rivaroxaban overexposure in these patients. On the other side, apixaban [77–81] and edoxaban [82] appear to be safe in patients with severe renal impairment; and apixaban has been already approved in patients with severe kidney dysfunction by the U.S. Food and Drug Administration. Thus, these agents might be considered (after serious assessment of risk/benefit ratio) in T2D patients with AF and severe renal insufficiency; but, as already stated, there is still a need for a study specifically examining the efficacy and safety profile of DOACs in patients with diabetic kidney disease for final conclusions and recommendations.

Finally, in fact, up-to-date, there is no larger prospective study specifically examining the effect of T2D on DOACs' efficacy, safety, and plasma levels/activity; and currently available data come from subanalyses of previously performed phase III clinical studies, registry analyses, or a small-sample prospective trial. In fact, phase III clinical studies with DOACs were not design to examine the effect of T2D on clinical outcomes in DOACs and warfarin-treated patients, and several T2D patients (for example, those with reduced glomerular filtration or those with prosthetic valves) were excluded from these trials [83]. Therewithal, due to the slightly different design of these trials, it is difficult to compare the T2D subalyses with one another to answer the question which of the DOACs is the most favorite for long-term anticoagulation in T2D individuals. Furthermore, there is no larger prospective study directly comparing the efficacy and safety of DOACs (dabigatran versus rivaroxaban versus apixaban versus edoxaban) in T2D patients. So, right now, it seems that the efficacy and safety of dabigatran, rivaroxaban, and apixaban (there is no study for edoxaban) is in T2D patients' comparability; but this might be changed if a study directly comparing these agents in T2D patients with AF will be performed. Thus, definitely, there is a need for further research regarding this issue.

## 8. Conclusion

Current evidence suggests comparable (or even more favorable) efficacy and safety of DOACs for long-term anticoagulation in T2D patients with AF. However, as the current evidence comes from subanalyses of phase III clinical trials and from limited amount of postmarketing analyses, further research is needed regarding this issue.

## Figures and Tables

**Table 1:** Pharmacologic profile of direct oral anticoagulants.

Parameter	Apixaban	Dabigatran	Edoxaban	Rivaroxaban
Target	Factor Xa	Thrombin (factor IIa)	Factor Xa	Factor Xa
FDA-approved indications	AF, VTE (treatment, secondary prevention, prophylaxis)	AF, VTE (treatment, secondary prevention, prophylaxis)	AF, VTE (treatment)	AF, VTE (treatment, secondary prevention, prophylaxis)
Evidence in T2D patients	Subanalysis of phase III trial [55], one postmarketing study [34]	Subanalysis of phase III trial [32], several postmarketing data [33, 34]	Limited data from phase III trial [57]	Subanalysis of phase III trial [46], several postmarketing data [33, 34, 47–50]
Acceptable safety profile in patients with severe renal dysfunctions	Yes (FDA-approved indication)	No (75 mg twice daily regimen approved only in the U.S.)	Yes (limited data)	No
Specific reversal agent	Andexanet alfa	Idarucizumab	Andexanet alfa	Andexanet alfa
Half-life (hours)	12	8–15	10–14	7–11
Renal clearance (%)	25	80	50	33
Dialysable	No	Yes	No	No
Prodrug	No	Yes	No	No
Bioavailability (%)	60	6	62	60–80
Time to peak effect (hours)	1–2	1–3	1–2	2–4
Drug interactions	Avoid apixaban with concomitant use of dual P-gp and moderate CYP 3A4 inhibitors	Dose reduce dabigatran with concomitant P-gp inhibitor; possible interaction with proton pump inhibitors	Avoid concomitant use of rifampin; no adjustments for concomitant P-gp inhibitors	Avoid rivaroxaban with concomitant use of dual P-gp and moderate CYP 3A4 inhibitors

AF: atrial fibrillation; CYP: cytochrome P 450; FDA: Food and Drugs Administration; P-gp: glycoprotein P; VTE: venous thromboembolism; U.S.: United States.

**Table 2:** Clinical outcomes in of entire AF patients and T2D AF patients from phase III studies with DOACs in AF.

Drug	Phase III study in AF	Number of patients (number of T2D patients)	Efficacy outcomes (entire population)	Safety outcomes (entire population)	Efficacy outcomes (T2D patients versus ND patients)	Safety outcomes (T2D patients versus ND patients)	References
Dabigatran	RE-LY	18,113 (4,221)	1.11 versus 1.69%/year, HR 0.66 (*p* < 0.001 for superiority) for high-dose dabigatran versus VKA; 1.53 versus 1.69%/year, HR 0.91 (*p* < 0.001 for noninferiority) for low-dose dabigatran versusVKA	3.11 versus 3.36%/year, HR 0.93 (*p* = 0.31) for high-dose dabigatran versus VKA; 2.71 versus 3.36%/year, HR 0.80 (*p* < 0.003) for low-dose dabigatran versus VKA	HR 0.61 versus 0.67 (*p* = 0.76) for high-dose dabigatran versus VKA in T2D versus ND group; HR 0.74 versus 0.97 (*p* = 0.24) for low-dose dabigatran versus VKA in T2D versus ND group	HR 1.12 versus 0.86 (*p* = 0.09) for high-dose dabigatran versus VKA in T2D versus ND group; HR 0.91 versus 0.76 (*p* = 0.26) for low-dose dabigatran versus VKA in T2D versus. ND group	[27, 32]
Rivaroxaban	ROCKET AF	14,264 (5,695)	1.7 versus 2.2%/year, HR 0.79 (*p* < 0.001) for rivaroxaban versus VKA	14.9 versus 14.5%/year, HR 1.03 (*p* = 0.44) for rivaroxaban versus VKA	HR 0.82 versus 0.92 (*p* = 0.53) for rivaroxaban versus VKA in T2D versus ND group	HR 1.00 versus 1.12 (*p* = 0.43) for rivaroxaban versus VKA in T2D versus ND group	[39, 46]
Apixaban	ARISTOTLE	18,201 (4,547)	1.27 versus 1.60%/year, HR 0.79 (*p* < 0.001 for noninferiority, *p* = 0.01 for superiority) for apixaban versus VKA	2.13 versus 3.09%/year, HR 0.69 (*p* < 0.001) for apixaban versus VKA	HR 0.75 versus 0.80 (*p* = 0.70) for apixaban versus VKA in T2D versus ND group	HR 0.96 versus 0.60 (*p* = 0.003) for apixaban versus VKA in T2D versus ND group	[52, 53, 55]
Edoxaban	ENGAGE AF-TIMI 48	21,105 (7,624)	1.18 versus 1.5%/year, HR 0.79 (*p* < 0.001) for high-dose edoxaban versus VKA; 1.61 versus 1.5%/year, HR 1.07 (*p* = 0.005) for low-dose edoxaban versus VKA	2.75 versus 3.43%/year, HR 0.80 (*p* < 0.001) for high-dose edoxaban versus VKA; 1.61 versus 3.43%/year, HR 0.47 (*p* < 0.001) for low-dose edoxaban versus VKA	HR 1.42 versus 1.52 (*p* = 0.54) for high-dose edoxaban versus VKA in T2D versus ND group; HR 1.9 versus 1.52 (*p* = 0.35) for low-dose edoxaban versus VKA in T2D versus ND group	HR 3.06 versus 3.94 (*p* = 0.70) for high-dose edoxaban versus VKA in T2D versus ND group; HR 1.74 versus 3.94 (*p* = 0.52) for low-dose edoxaban versus VKA in T2D versus ND group	[57]

AF: atrial fibrillation; ARISTOTLE: Apixaban for Reduction in Stroke and Other Thromboembolic Events in Atrial Fibrillation; DOACs: direct oral anticoagulants; ENGAGE AF-TIMI 48: Effective Anticoagulation with Factor Xa Next Generation in Atrial Fibrillation-Thrombolysis in Myocardial Infarction 48; HR: hazard ratio; ND: nondiabetic; RE-LY: Randomized Evaluation of Long-Term Anticoagulation Therapy; ROCKET AF: The Rivaroxaban One Daily Oral Direct Factor Xa Inhibition Compared with Vitamin K Antagonism for Prevention of Stroke and Embolism Trial in Atrial Fibrillation; T2D: type 2 diabetes; VKA: vitamin K antagonist.
